# Modest doubt: enabling discovery across maritime heritage records

**DOI:** 10.1007/s42803-025-00117-5

**Published:** 2026-03-02

**Authors:** Jack Pink, Shrikriti Singh, Adriane Chapman, Fraser Sturt

**Affiliations:** 1 Environmental Social Sciences and Stanford Robotics Center, Stanford University, Stanford, CA United States of America; 2 Department of Archaeology, University of Southampton, University Road, Southampton, Hampshire SO17 1BJ United Kingdom; 3 Electronics and Computer Science, University of Southampton, University Road, Southampton, Hampshire SO17 1BJ United Kingdom

**Keywords:** Maritime archaeology, Few-Shot learning, Named entity recognition, Metadata enrichment

## Abstract

The United Kingdom’s Maritime Heritage records hold vital information on heritage assets and archaeological contexts key to addressing current research priorities. These records stand alone, rarely connecting, demanding an arduous and time-consuming process of investigation and cross-referencing for synthesis to occur. Challenges intensify due to the qualities of this data; they stem partly from legacy information not initially intended for heritage recording purposes. Complications also arise from the varied methods of recording, storing, and disseminating this information deployed over time. However, archaeologists now possess Named Entity Recognition (NER) and Natural Language Processing (NLP) tools with the potential to organize and search such fragmented databases. This paper explores a method to enhance and enrich these datasets by employing Few Shot learning techniques to perform Multiclass Text Classification. The Welsh National Monuments Record (NMR), a glimpse into the U.K.’s maritime heritage, has been utilized to test these approaches.

## Introduction

Archaeology, like many other areas in the heritage sector, is attempting to address the challenges born of a long and complex history of data generation as well as distributed and fragmented storage. A record that should transcend national boundaries to create narratives of our shared past is all too often defined by histories of data encoding and management. Steps have been taken to address this challenge, with the Ariadne project (Meghini et al., 2017) (accessible via https://www.ariadne-research-infrastructure.eu) being notable for its scale, scope and ambition. However, significant gaps remain, with records related to maritime heritage (wrecks, infrastructure, and submerged landscapes) often proving elusive or lacking detail (being ‘thin’ in nature). In this paper we present work that looks to address the challenges of integrating fragmented and heterogeneous national maritime datasets to create a broader transnational picture.

The United Kingdom’s maritime heritage records are critical assets. They are central to key decision-making processes; from shaping the placement and development of national infrastructure through to enabling access to, and ensuring effective protection of, sites. With increasing use of maritime space for delivery of renewable energy to meet net zero demands (Putuhena et al., 2023), and the hosting of data and communication infrastructure, this information is becoming more important and more frequently used as part of the planning process. These use cases coincide and run parallel to broader scholarly and public interests, from research into wrecks, coastal towns or submerged landscapes.

Within the UK, the National Heritage Records are split across four agencies, one for each devolved nation:

- England - Historic England (HE)
- Northern Ireland - Department for Communities (DfC)
- Scotland - Historic Environment Scotland (HES)
- Wales - the Royal Commission for the Ancient and Historic Monuments of Wales (RCAHMW)

Records are stored in different ways and follow different data conventions. There is no unified record, nor an easy means to collate or search within or across datasets. This hampers attempts to understand the nature of the record, be it for planning or academic purposes. Thus, key questions remain beyond our grasp. How does the record change through time? What are the spatial characteristics of wrecking for different periods and ship types? Where are our most significant wrecks located? How would a non-specialist access this information?

This paper sets out the first steps taken to address these issues. It charts the creation of a linked open data source for the United Kingdom’s maritime heritage, leveraging Natural Language Processing (NLP) and Few Shot Learning (FSL) tools to enable harmonisation. The tools created and lessons learned resonate beyond this specific example and extend to numerous different agencies and institutions, where fragmented data represent a challenge to academia and industry.

## Problem statement

Records relating to maritime heritage within the UK are divided across the four national agencies, commercial and non-governmental organisations (such as the United Kingdom Hydrographic Office - UKHO, Lloyd’s Register, and National Maritime Museum), as well as numerous paper archives. These records have been generated over hundreds of years, for different purposes. Furthermore, each of the national agencies has created their own metadata record for key sites. While these records share common fields (e.g., location, name, additional information) key details such as ‘craft type’ are often subsumed within long text entries in a ‘description’ field. Ultimately, these datasets sit as individual silos of knowledge that do not communicate with each other and are ordered and structured differently. The National Monuments Record (NMR) for Wales is a good example of this. The dataset itself is rich, with significant information held within the description field, but little information separated out into other fields, hampering search and linking activities (see Table 1). Those records often contain exactly the same information as the historic source they are derived from. These sources were never intended to be an historical record and were instead records associated with areas like insurance or safety at sea. They therefore contain the same biases and priorities and can omit information that would be considered essential today. Enrichment of the metadata using text classification to extract key terms from rich text descriptions is the first step to enhance the dataset; here individual maritime craft types are focused on. This enhancement will aid in the search and interrogation of entries by allowing users to identify the type of ship being dealt with.

**Table 1:** An example of a shipwreck record from the Coflein database showing all the data fields present (table by the authors)

NPRN	NMRW Name	Description	NMRW Heritage Resource Type	NMRW cultural period	NMRW evidence	OSGB Grid Ref.	Geometry	Community	Unitary Authority	Old County	ENTRY DATE	ENTER BY	LAST UPDATE	UPD BY
272340	Abbey	“This record consists of a documentary reference to a shipping casualty which has been assigned to the maritime named location HOLYHEAD HARBOUR pending more information which may allow a more precise location for the loss to be assigned. Event and Historical Information: The ABBEY was a wooden sailing vessel. At time of loss on 30 August 1819, it was carrying a cargo of china clay from Charleston, Devon, to Liverpool under the command of master Cowling. The vessel is reported to have run onto the back slope of the pier at Holyhead and to have been bilged. Sources include: Larn and Larn Shipwreck Database 2002 Lloyds List, 21 September 1819, issue number 5423 Maritime Officer, RCAHMW, June 2008.”	WRECK	Post medieval	Documents	SH2568882760	GEOMETRY COLLECTION (POINT (-4.6179977 53.313004))	Maritime	Maritime	Maritime	20/09/2006	RC_WALES	26/03/2010	“DMG”

## Scholarly context and the state of the art

This is not a new problem. Archaeology has been profoundly aware of issues relating to data storage and fragmentation for at least three decades. Larsen (1992, p.3), in the preface to a volume dedicated to national archaeological records, noted that:

“Ever increasing amounts of money are being turned towards the task of making the manual, archaeological archives accessible through new technology. The reason for this must be sought in increased and accelerating physical development and land exploitation”

Within the same volume Aberg and Leech (1992) provide an account of the national archaeological record in England. They note that the 1980s had been a period of data capture and the 1990s should be one focused on how to link disparate datasets and make the data more widely known and available (Aberg & Leech, 1992, p. 165). While the aspiration was there and efforts were made to draw datasets together and increase use, the ultimate goal of meaningfully connecting records was not reached. Richards (2006, p.199) highlighted these issues and built on the work of Rahtz (1986), stating the need to resolve the challenge of broadened access and participation whilst guiding readers/participants through a coherent story and avoiding “drowning in data”. In this he recognises that in the 20 years since these issues were first raised those questions had not been answered. Instead, we can see that datasets and records developed an increasingly digital footprint, but not necessarily a more coherent one, as more and more information was digitised and recorded online.

**Fig. 1:**
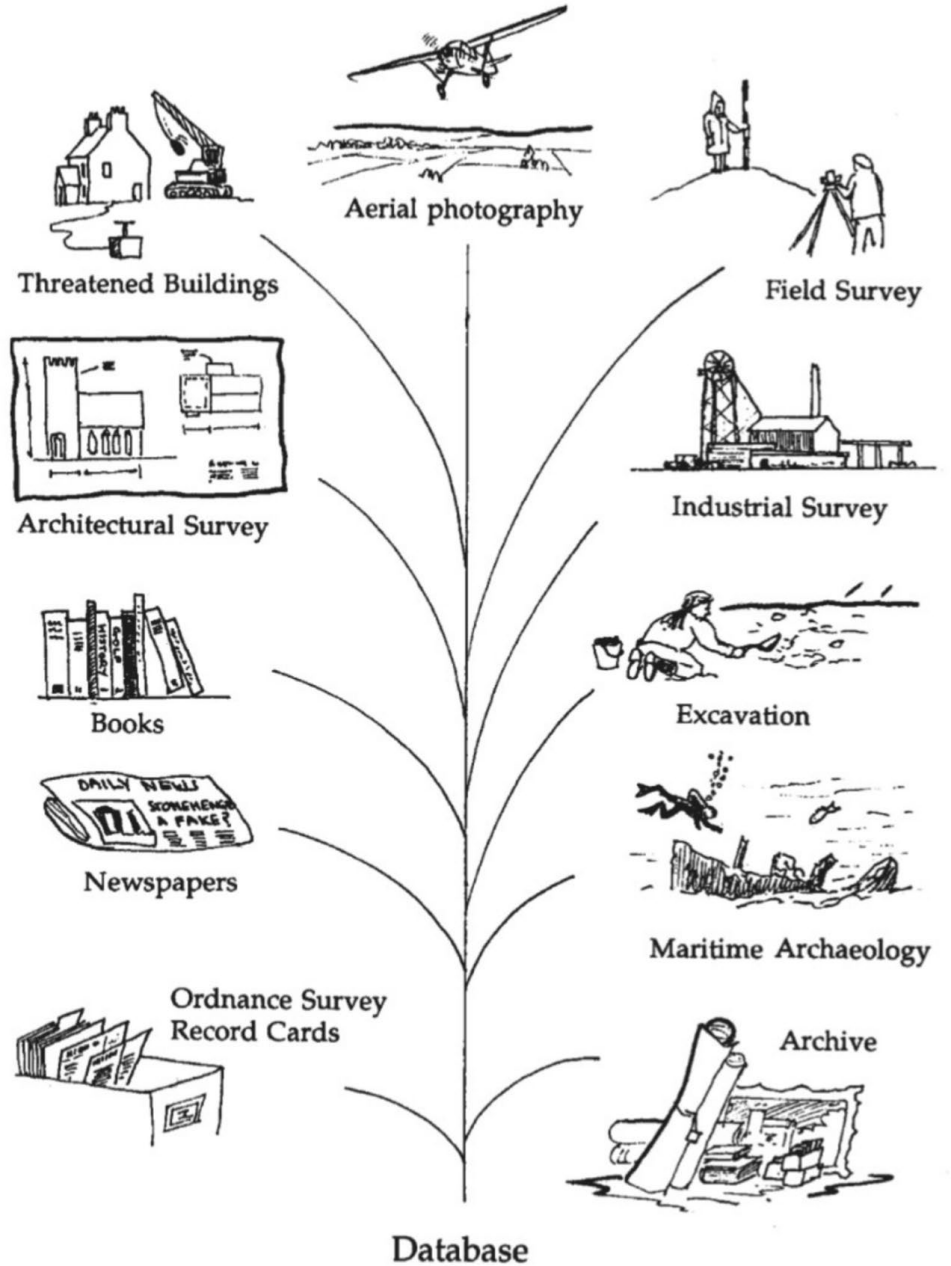
The use of GIS at the Royal Commission on the Ancient and Historical Monuments of Scotland (RCAHMS) to integrate datasets from within the organisation’s records (taken from (Lock, 2003, p.208), Figure 6.6, after Murray 1992)

In a partial answer to calls for an increasingly digitised record and more online records, Geographical Information Systems (GIS) became widely utilised to both hold and display heritage datasets (Lock, 2003, p.165–7). There is a depth of literature on the use and deployment of these systems for a variety of purposes (e.g., De Roo et al., 2013; Harris & Lock, 1995; Wheatley & Gillings, 2013), and they certainly resolved the need to store and order data in a way that addressed the questions asked by Rahtz (1986), Aberg and Leech (1992), and Richards (2006). However, these deployments became knowledge silos, isolated from one another and organisationally distinct in their structure and ordering. The primary focus of such systems was linkage of internal resources (see Fig. 1), not external linkage to one another (Lock, 2003: 207-11; Backhouse, 2006: 48-49). Through the 2010s the architecture that would have enabled such linkage began to appear and be utilised. However, usage also began to disappear, pointing to the fragility of such dependencies. This is exemplified by the British Museum’s SparQL Endpoint (Datahub, n.d), a very promising start for linked open data which was summarily removed. By the mid 2010’s there was very little uptake of available architectures to facilitate the connection aspired to in the 80s, 90s, and 2000s.

The core of those records is text-based, and text analysis is directly relevant to the humanities as a whole, not just archaeology. Within the Digital Humanities (DH) text analysis has developed from classic Machine Learning techniques like Support Vector Machines, Naive Bayes, and decision trees that can be used to classify text or grouping texts into sets based on similarities in the data they contain, to off-the-shelf Natural Language Processing tools, like spaCy or Gensim that lend themselves to parsing and analysing textual data. More recently solutions that deploy Deep Neural Networks (DNN) (like Google’s SentenceBERT) as discussed in Suissa et al. (2022a). have come to the fore. The advantage of DNNs to the Digital Humanities is their applicability to Semantic Analysis (understanding the context and meaning of text), Entity Recognition and Entity Linking (identifying entities, such as people, places, or dates within a text and establishing connections between them), and for tasks like translation or analysis of datasets in multiple languages. There are challenges to the deployment of DNNs, the most relevant to this paper being the ‘(un)availability’ of training data (Suissa et al., 2022a, 269 and 271). Within the Digital Humanities, Maritime Heritage Data occupies a small niche, and it is one that lacks datasets that are clearly labelled with ‘right’ and ‘wrong’ examples of key text analysis tasks. As such, any acitivity in this space requires some level of additional work to generate a dataset suitable for training the model.

Against this backdrop and developing suite of tools Archaeology has continued to explore the application of Information Extraction (IE). This has been for two primary purposes:

1. To better integrate fragmented records.
2. To leverage information from ‘grey’ literature.

Within projects exploring IE for heritage data, approaches such as NER and multiclass text classification have been identified as a powerful tool. NER has been successfully deployed in searches and tasks related to ‘grey’ literature; in Archaeotools (Jeffrey et al., 2009; Richards et al., 2011), STAR (Tudhope et al., 2011), STELLAR (Binding et al., 2015), ARIADNE (Meghini et al., 2017) and most recently AGNES (Brandsen et al., 2022).

To date there has been no application of NER systems to the records of the National Heritage Agencies of the UK or its equivalents found elsewhere. The closest piece of work is the ARIADNE+ project that enables European records to be searched via an Open-Linked dataset (Meghini et al., 2017). ARIADNE+ serves as useful reference point as it is the current state-of-the-art for disseminating archaeological knowledge through a unified repository. The Heritage Record in the UK is already a widely accessible resource, as these records have been digitised and are accessible through a range of portals e.g., the Historic Environment Records (HER) in England (HistoricEngland, 2012)or the individual NMRs in the devolved nations (e.g., RCAHMW, 2023). Furthermore, work has already been undertaken by the responsible Heritage Management Agencies and the UK’s Archaeological Data Service to move towards principles of Findability, Accessibility, Interoperability, and Reusability (FAIR) (Wilkinson et al., 2016). An approach that is better placed to produce a linked dataset. However, there is still a degree of disarticulation between each of the four devolved NMRs and some of the data within relies on thin metadata despite having rich detailed description fields. Recent work focusing on archaeological grey literature highlights the potential for IE and NER to enhance datasets for use in archaeological research (Richards et al., 2015; Vlachidis & Tudhope, 2016). However, many of the same arguments hold true for NMR-type data and its relationship with other maritime heritage datasets. The presence of the rich-text descriptions is the key factor, as IE from these fields will enable enhancement of the NMR metadata.

The works of Brandsen et al. (2022), and Vlachidis and Tudhope (2021) represent the state of the art in the application of NER extraction techniques in archaeology, and provide extensive reviews of previous work. The approach outlined in this paper relies heavily on domain expertise and ‘human-in-the-loop’, which have been shown to be effective elsewhere (e.g., Chun & Elkins, 2023). The need for a clear structured vocabulary when conducting this form of work has been explored by Vlachidis and Tudhope (2016) and this lesson is observed in the work done for this paper. Semantic Annotation using text classification or NER to enhance metadata enables not just a word matching exercise but a search for entities and relations (Vlachidis & Tudhope, 2016, p.1140). However, the datasets within any repository must have sufficiently rich metadata to support this.

Metadata creation remains a significant challenge for archaeology. The process can be time-consuming, repetitive, boring, and inconsistent when done manually, particularly if the critical importance of its quality is not stressed or understood. Middle (2023) has shown the key of role documentation for digital systems in the reproducibility and usability of digital tools. It is also important to ensure that the result of any metadata creation or enrichment activity is properly integrated with wider archaeological data (Vlachidis & Tudhope, 2021, p.4-5). The approach in this paper directly addresses metadata enrichment and its integration into a searchable system. It looks to resolve the challenges that emerge out of past practice, where long form detailed information was fed into a ‘description’ field, but other fields were inconsistently populated or ignored. This is a unique contribution of the work done here. This enrichment is performed by a Few-Shot system leveraging human-in-the-loop to tag individual records by type.

Brandsen’s AGNES project (Brandsen et al., 2022) provides a clear outline of the pitfalls and problems that the application of NER to heritage data can raise. The exploration of these issues also highlights the main challenge facing the deployment of NER, that of bias. Studies of bias in AI systems seem to occupy a niche, they are certainly not as interwoven into the deployment of such systems as one might expect. The work of Crawford (2021) performs its titular role as an atlas for these issues. Much of the work cited by Crawford, and for other key texts such as those by Benjamin (2019), Bowker and Star (2000), or Noble (2018), focus on the deployment of categorisation in respect to people. Haraway (2013, p.163 and 164) makes the point that there has been a *“search for a common language”*, and that same language works to enforce meaning and enable control in a system. This is relevant to the domain of archaeology and to NER deployment on heritage datasets even if those datasets are primarily text—rather than image—based. The clearest link is the discussion of classification and how reliant AI algorithms are upon skewed and incomplete datasets. Approaching the examples and discussions within each of these pieces of literature a few key points become apparent:

- Deployment of AI systems, relies on data being provided to the model from which it can learn a response. That data must be classified and sorted for a model to recognise entities and context.
- Classification relies upon fitting data, things, or people into pre-existing criteria. New criteria can be created for new entities however these do not account for every variation, difference, or all the details of an entity. Categories, at best, fit where they touch.
- Many of the discussions and debates currently swirling around the topic of AI and Classification mirror discussions held within Archaeology as a discipline with decades of experience dealing with the tangled issue of typology, classification, and sorting (e.g., Baines & Brophy, 2006, 212-213). By its very nature archaeology is a subject concerned with things and people and understanding their context.

From these points it is necessary to explore some of the further ideas raised by (Crawford, 2021). The world of Digital Heritage relies on a system of simulacra, or stand-ins for the materials, documents, and people being explored. The map is not the territory, the record about a shipwreck is not the shipwreck. A similar conversation took place in the early 2000s, where archaeological data, both digital and non-digital, were recognised not as essential truths about the past, but instead as a result of archaeologists choosing how to describe the world (Baines & Brophy, 2006, p.211). It is impossible for the NER systems being discussed by this or any other paper in archaeology to directly engage with the material culture the records are concerned with, only their representations.

At present the only way to achieve this is the creation of a digital copy, a “Digital Twin” (Niccolucci et al., 2022, p.4-5). Unfortunately, those digital twins cannot capture the infinite variety and detail of even a single artefact, document, or person – they remain a simulacra. This is problematic, within the literature there appears to be a thread that sets Digital Systems (including linkage and AI) and the data they work with as the authoritative record (e.g., Roosevelt et al., 2015, p.339-40). For the dataset targeted by this project this is an issue, as the extraction of terms relies on records derived from historical sources containing bias as a matter of course. A product of their age and original purpose, which was not to serve as a permanent record of an individual ship or event. It is likely that the records will reflect the concerns and focus of the time, for example 19th century casualty returns will be mainly concerned with ship names and utilise named locations rather than specific co-ordinates to locate losses. Those Named Locations present a further problem as they are highly subjective. They can overlap one another or have more than one name for the same place. This adds a degree of vagueness to location data, and certainly has contributed to a lack of certainty when locating shipwreck sites. This is an example of the kind of challenges the older parts of the NMRs contain. This paper outlines a text classification approach to tag records thereby enhancing the utility of NMR records.

In summary, this paper presents a case study that utilises a record with a known, albeit problematic, provenance. The tagging undertaken utilises a recognised Heritage Standard, the Forum on International Standards in Heritage (FISH) Maritime Craft Thesaurus (HeritageStandards, 2023). The resulting dataset has been lodged with the RCAHMW and made available to the public via https://unpathd.ads.ac.uk. At no point is the suggestion made that what is performed here represents the final step with regards to enriching thin metadata. It is a time-saving tool that allows for best-fit contextual labelling of text-records, therefore enhancing the criteria that can be identified through search functions.

## The data

**Fig. 2:**
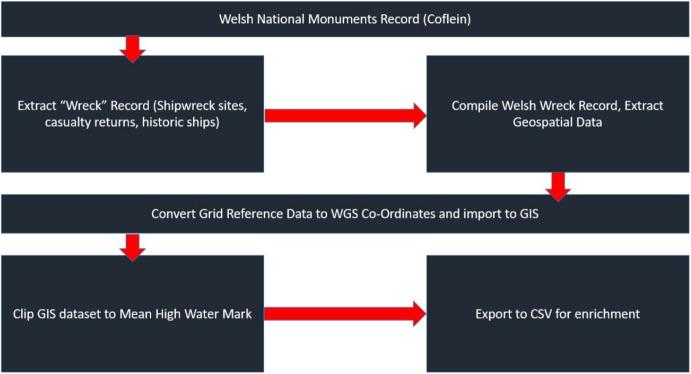
An example of an extraction process for NMR data, this same pipeline can also be used to extract records from UKHO shapefiles (image by authors)

The primary text dataset comprised the National Monuments Record for Wales supplied by the RCAHMW. The data used by this project can be accessed via: https://datamap.gov.wales. A live version of individual records can be accessed through the Coflein website (RCAHMW, 2023), so hereafter called “Coflein” and consists of both maritime and terrestrial records. The data used in this paper was a subset extracted from the broader database, reflecting those records found in the marine zone (see Fig. 2). Some shipwreck entries contain much more detailed information than others. That variety reflects the history of the U.K.’s NMRs. These have been compiled over decades, by multiple different individuals who all approach the task differently, sometimes including information that was extracted from casualty returns in the 19th century and is otherwise unchanged from that first publication. The thesaurus used for tagging is a curated list of terms managed through FISH (HeritageStandards, 2023) and contains 294 individual craft types. This list represents a definitive vocabulary for maritime craft and is therefore an ideal resource for the extraction of terms from the Coflein dataset.

The challenge presented by Coflein is that much of the record predates the establishment of FISH or any other definitive vocabulary list. This means that some entries within the Coflein record will not contain any term that directly connects to the entries in FISH. It is this disconnect that created the opportunity for an NLP-based approach to identify craft types based on context within the long-form text of each entry’s description. In addition by training the model with the basic FISH craft types it is kept applicable to all the National Records and appending regionally specific terms (such as “Birlin” for HES) is then a straightforward task.

**Fig. 3:**
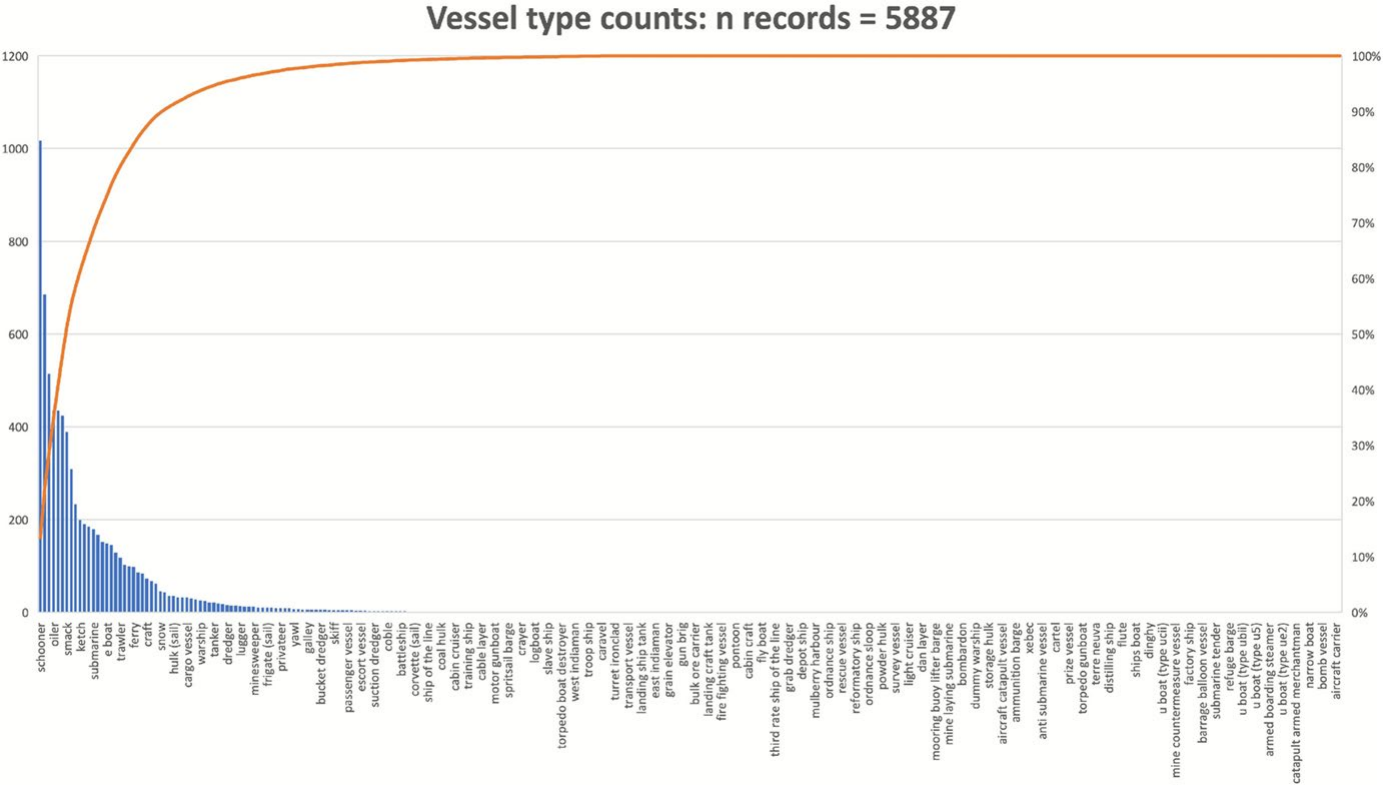
Chart showing the number of examples for each FISH maritime craft thesaurus entry within the Coflein database (image by the authors)

A subset of data was manually annotated by ship type. This consisted of the first ten entries for each ship type. However, for most of the craft types there were less than ten entries for that type, in these cases all the annotated records for a particular ship type were used (see Table 2). Many had only one example in the entire dataset. That subset of data was annotated by a single domain expert. The expert manually tagged the correct maritime craft in each individual record based on the information in the “Description” field. The tag was created using one of the types from the FISH Maritime Craft Thesaurus (HeritageStandards, 2023). The result was not a balanced spread across the different types with some represented by many examples and some with one or even none. In total, that annotated dataset comprised 634 records extracted from Coflein with a minimum of one record for each craft type. Most of the craft types within the FISH Maritime Craft Thesaurus were not represented in Coflein, there were a total of 99 unique craft types from the thesaurus represented by the labelled data. This difference to the thesaurus can be seen most clearly in Fig. 3 where the presence of a term in the rich text description is visualised against the full thesaurus. The labelled dataset was split into ‘train’ and ‘test’ sets in a 70:30 ratio, this split is stratified to ensure that the proportion of samples in each class of the target variable is maintained across the training and testing sets.

**Table 2:** Number of labelled records for each craft type within the labelled data (table by the authors)

Craft type	Number of annotated records
Amphibious vehicle	1
Anti submarine vessel	2
Barge	14
Barque	55
Barquentine	5
Bawley	2
Bilander	4
Boom defence vessel	1
Brig	88
Brigantine	22
Cargo vessel	68
Catamaran	3
Clipper	2
Coaster	15
Collier	6
Corvette	1
Crane barge	1
Cutter	30
Destroyer	2
Dredger	8
Drifter	8
Ferry	11
Fishing vessel	25
Flat	3
Flyboat	1
Frigate	1
Galliot	2
Glass bottomed boat	1
Grimsby	1
Ice breaker	1
Iron lighter	1
Ketch	13
Launch	2
Lifeboat	5
Lighter	4
Liner	1
Longliner	1
Lugger	11
Motor barge	1
Motor boat	2
Motor launch	1
Motor tug	3
Motor vessel	2
Naval vessel	5
Paddle steamer	4
Pinnace	1
Pleasure craft	3
Pontoon	2
Pram	1
Punt	1
Racing yacht	1
River tug	1
Rock cutter	1
Rowing boat	1
Sailing barge	1
Sailing vessel	14
Schooner	105
Scow	1
Seaplane tender	1
Ship of the line	2
Skiff	1
Slave ship	1
Smack	9
Store ship	1
Tanker	3
Tender	3
Trow	2
Tug	5
Unidentified vessel	12
Warship	1
West indiaman	2
Whale processing ship	1
Wherry	1
Yacht	8
Yawl	8

**Fig. 4:**
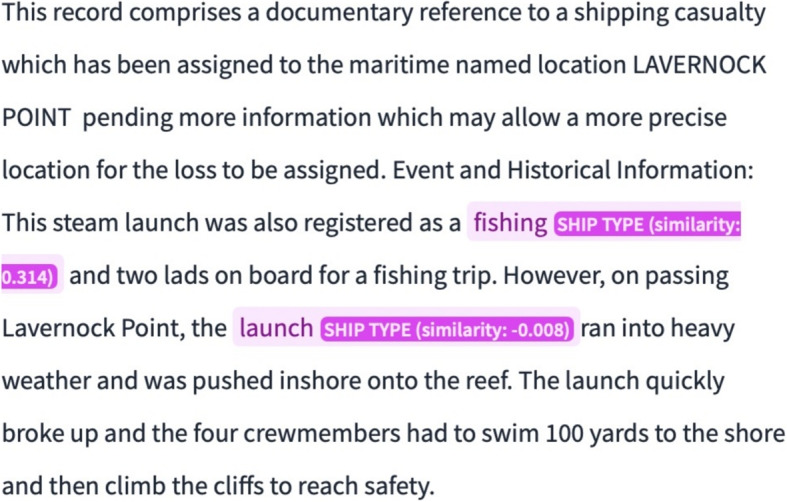
Example showing text tagging during text classification (image by the authors)

Finally, some entries in the NMR have no information within their text that identified a ship type, other entries had more than one entry (see Fig. 4). This informs on the quality of the information within the record and highlights the variance that can exist between individual records. In some cases, records relate to fishermen’s fastenings or seafloor features that have no associated historical or archaeological identification. Until these sites and locations are directly investigated in the real world there is no means to enrich the record associated with them.

## Methods, material, and approaches

This is a relatively new task in archaeology, so techniques are being deployed that have worked in other communities to understand what is appropriate for our domain. Several different technological approaches have been explored, including classic supervised machine learning approaches, shot-learning approaches, and application of a rule-based system. In the deployment of the models the aim was to identify the best model, based upon the input data available, that could then be deployed over a maritime heritage dataset it had never encountered before. Creating a model with this functionality makes it possible to enact enrichment and extraction tasks over any of the UK’s NMRs.

### Multiclass text classification

**Fig. 5:**
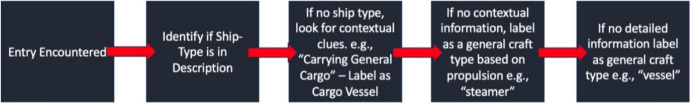
Flow diagram to represent the process used by domain experts to label craft type from Coflein records (image by the authors)

Labelling of data was undertaken by the team’s Maritime Archaeology domain expert and leveraged a formal method to establish which craft type related to each entry in the dataset (see Fig. 5).

#### Shot learning overview and approaches

In Zero Shot Learning (ZSL) an AI model learns to recognize and categorize new, unseen classes based on their understanding of relationships between known classes. Instead of explicit training, models utilize semantic associations between tokens (word-parts) to make predictions for novel instances. This enables systems to bridge the gap between familiar and unfamiliar concepts, expanding their capabilities.

**Fig. 6:**
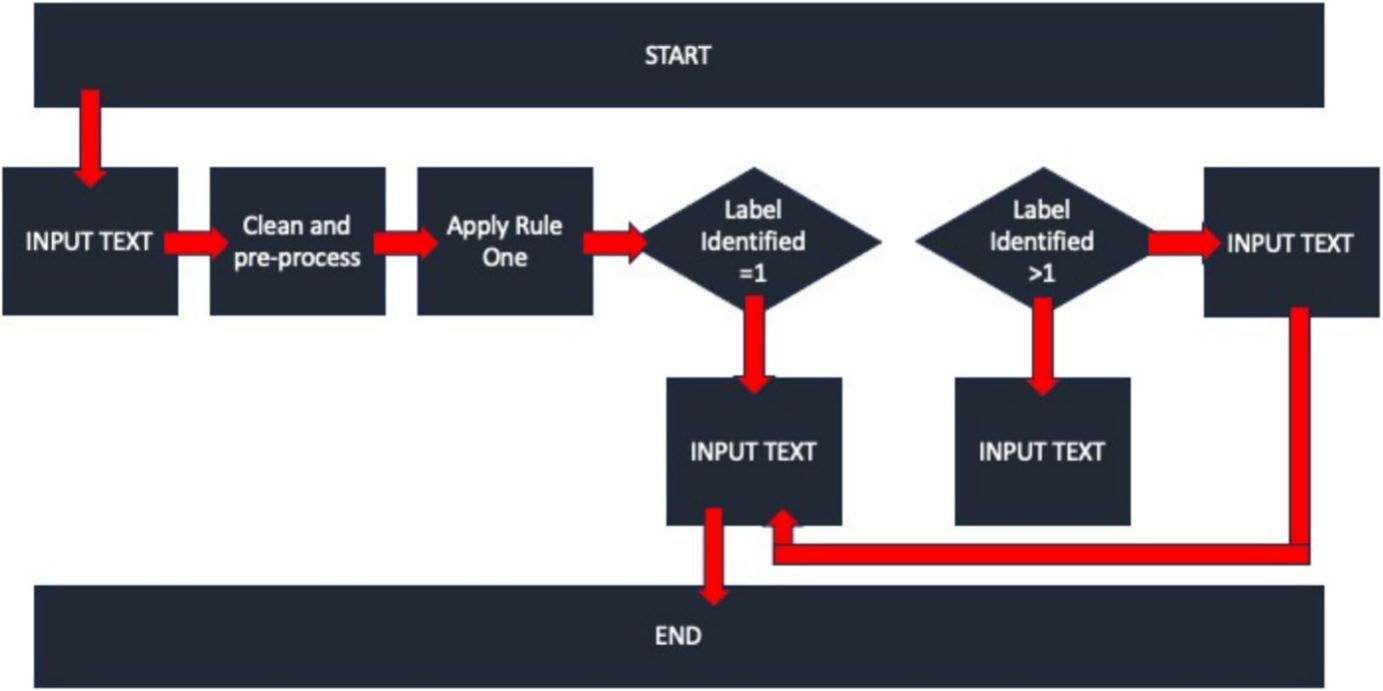
The complete ZSL loop including a cleaning phase and the rule-based split to process individual records (image by the authors)

For the ZSL approach in this paper the data can be split into two kinds, records where the craft type is present in the rich text description, and records where type could be summarised from the description using domain expertise. In the cases where there is no ship type present in the text the record was passed to the ZSL algorithms (see Fig. 6). A series of rules were created to be used with the ZSL approach based on the expertise of the team’s maritime archaeologist to follow the process they would use to identify the craft type a record would belong to (see Table 3). The similarity scores referenced by Rule 2 are included as Table 4. Two ZSL implementations have been used. The first is trained on the Sentence Transformers repository (Reimers & Gurevych, 2019) and maps the text to a 384 dimensional dense vector space. This finds the cosine similarity between each label and the text independently. The final prediction is the label that has highest similarity score. The second is trained on the MultiNLI dataset (Williams et al., 2017). This method deploys softmax classifier and distributes probabilities over all the labels. The label with highest probability values is returned.

**Table 3:** The rules used prior to engaging any models and their outputs (table by the authors)

Rule	Description	Output
Rule 1	Matches all ship-type labels present in the text and returns a list. If only one label is present this is marked as a strong match. If more than one label is present it is sent through to the ZSL algorithms.	Ordered list of ship type labels present in text.
Rule 2	If similarity scores are identify more than one label in an entry and a single preference cannot be identified preference is given to the label that occurs first in the sentence.	Ordered list of labels based on similarity and word location
Rule 3	Builds upon rule one. This tags labels returned by Rule One as nouns, spacy’s pre-implemented library is used for parts of speech tagging.	Ordered list of labels based on use of ship type name as noun (avoids erroneous tags from verbs that match ship types such as: sailing, fishing and so on)

**Table 4:** Similarity scores for sentence BERT ZSL and Facebook BART MNLI

Label	**Sentence BERT ZSL**	Facebook BART MNLI
Tanker	0.29562	0.00527
Trawler	0.24494	0.0034
Schooner	0.23405	0.00472
Submarine	0.23026	0.00338
Barque	0.20950	0.00446
Dredger	0.20717	0.0037
Naval	0.20137	0.03228
Barge	0.20076	0.00321
Tug	0.19190	0.00439
Liner	0.17651	0.00649
Sloop	0.14961	0.00644
Brig	0.14320	0.00232
Yacht	0.13979	0.39251
Ketch	0.11566	0.00417
Lighter	0.11265	0.02292
Craft	0.10871	0.28454
Wherry	0.10382	0.01546
Yawl	0.10137	0.01307
Lugger	0.09451	0.0063
Drifter	0.08355	0.01842
Cargo	0.05608	0.0112
Fishing	0.05578	0.00352
Launch	0.054143	0.01809
Smack	-0.003999	0.02922
Transport	-0.00848	0.03024
Patrol	-0.018542	0.00987
Customs	-0.03886	0.02121
Indiaman	-0.056150	0.00285
Escort	None	0.01245
Passenger	None	0.00548

#### Human-in-the-loop (HiL) approach

**Fig. 7:**
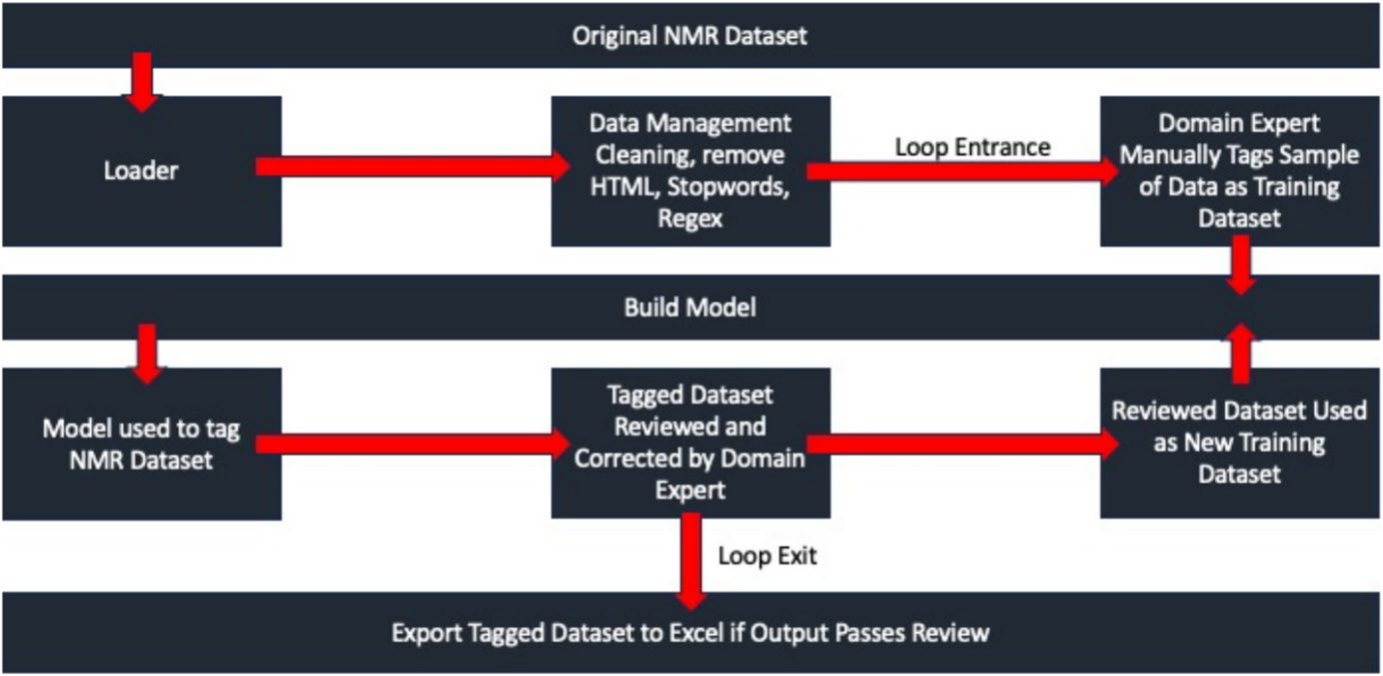
The human in the loop data preparation and model fixing (image by the authors)

The phrase “Human-in-the-loop” refers to the involvement of a specialist or domain expert in an AI-system. Literally a human performing a task in the automated loop of tasks (see Fig. 7). This involves a continuous cycle of human input and machine processing. Humans review and guide the output of AI algorithms, correcting errors and providing insights. This iterative feedback loop refines algorithms over time, enhancing their performance. This approach is particularly valuable in tasks like data annotation, model validation, and decision-making, combining the efficiency of machines with human intelligence, ensuring accuracy, adaptability, and ethical considerations in complex and evolving scenarios. In this case an FSL model was deployed and a domain expert was utilised to generate the labelled dataset. That expert was then tasked with correcting predictions from the model.

#### BERT and simpletransformers

BERT or Bidirectional Encoder Representations from Transformers (Devlin et al., 2018) is the main model in this paper. This was the same choice made for AGNES (Brandsen et al., 2022). We have used BERT-base-cased with the simpletransformers (Rajapakse, 2020) library’s ClassificationModel class to define and train the model. These are used for for sentence/text-level classification (multiclass text classification). Prior to BERT, only left-to-right or right-to-left directional models were used, which sequentially read the words. To determine the context of each word based on its surrounds, BERT reads all the words at once. It goes through sentences pairwise to resolve semantic search. When there are hundreds or thousands of sentences this results in n(n - 1)/2 computations. This makes the model inefficient when it comes to semantic comparisons.

Few Shot Learning (FSL) enables a model to learn and make predictions from a small number of examples. It is particularly useful in situations where data is limited or expensive to obtain. One popular approach to FSL, is to use a pre-trained model, fine-tuned on a small number of labelled examples from the new task.

#### Summary of alternate approaches

Several conventional supervised learning approaches were also trialled for comparison, including Support Vector Machines (SVMs) (Cortes & Vapnik, 1995) and Random Forest (Breiman, 2001) classifiers implemented with TF-IDF and GloVe (Pennington et al., 2014) embeddings. These models were selected to benchmark performance against more recent Few-Shot and Zero-Shot learning architectures. While they provided useful baselines, their overall accuracy and F1 scores were substantially lower than the Few-Shot Learning (FSL) model with Human-in-the-Loop refinement. As such, their detailed configurations and results are not reported here, but their inclusion confirmed that transformer-based approaches offer the most effective performance for this dataset.

### Validation of methods

#### Accuracy

In order to establish the overall correctness of the model’s predictions, accuracy is measured as follows. The labelled dataset has been split into “Train” and “Test” sets on a 70/30 ratio. Accuracy is determined by comparing the model’s predictions on the “Test” set to the actual labels in the “Test” set after being trained on the “Train”. During the evaluation phase, the model makes predictions on the evaluation dataset. The accuracy is calculated by the total correct predictions out of all predictions.

#### Precision and recall

In order to understand what types of errors our methods have generated, we also measure precision and recall. To calculate precision and recall, for every prediction, we update the count of the following based on the prediction’s correctness or error type: True Positives (TP), False Positives (FP), True Negatives (TN) and False Negatives (FN). Precision considers false positives and is calculated via: $TP / (TP + FP)$. Recall considers the impact of false negatives and is calculated via: $TP / (TP + FN)$. F1 is a blend of precision and recall as: $2 * (Precision * Recall) / (Precision + Recall)$.

## Results and discussion

All the approaches discussed in the Methods, Materials, and Approaches section were applied to the data described in the Data section. However, FSL with HiL outperformed all the other models by such an extent that we present only the results from FSL with HiL and standard supervised learning approaches within this section (see Table 5). The results from the other systems were so poor they add little to the conversation after their implementation is discussed.

### Few shot learning results

The Few-Shot model, BERT with simpletransformers, performed well Table 5. The training dataset comprised hand-labelled data. The successful implementation based off this kind of training data demonstrates how powerful FSL can be when combined with human domain expertise in this context. The model’s accuracy was not impacted by the number of labels it was tasked with, indicating the model is robust and promising.

**Table 5:** FSL results from implementations of BERT using different numbers of data samples (table by the authors)

Data type	Model	Epoch	Batch size	Accuracy	F1 (Macro)
Data with at least 4 samples each	BERT	45	4	83.85	0.88
Data with at least 2 samples each	BERT	45	3	75.59	0.82

The model used for FSL classification was fine-tuned on 70% of the dataset before testing. Some ship types like ‘Wherry’ had only one entry in the entire RCAHMW dataset. The algorithm took just over one minute to train and each prediction took about 0.5 seconds. Precision and recall are shown in Table 6, where ship types had too few entries it was not possible to report precision and recall.

**Table 6:** Precision, recall and F1 scores for a selection of maritime craft types from the FSL model (table by the authors)

Ship type	Precision	Recall	F1 score
Barge	1	1	1.000
Barque	1	1	1.000
Brig	0.8	1	0.889
Cargo	1	1	1.000
Craft	1	0.667	0.800
Dredger	1	1	1.000
Drifter	1	1	1.000
Fishing vessel	0.667	1	0.800
Indiaman	0	0	None
Ketch	1	1	1.000
Launch	None	None	None
Lighter	1	0.5	0.667
Liner	None	0	None
Lugger	1	1	1.000
Naval vessel	1	0.4	0.571
Schooner	1	0.8	0.889
Sloop	1	1	1.000
Smack	1	1	1.000
Submarine	0.8	1	0.889
Tanker	1	1	1.000
Transport	0.125	1	0.222
Trawler	1	1	1.000
Tug	0.857	1	0.923
Wherry	None	None	None
Yacht	0.667	1	0.800
Yawl	1	0.667	0.800

Shot-Learners perform significantly better when they are fine-tuned, even though they are designed to generalise on unseen domains. The results also show only FSL could map synonymous words. In the craft list the word *“barque”* is the best example as it is synonymous for the word *“bark”*. Both words refer to a sailing vessel with square-rigged fore- and mainmasts and a fore-and-aft rigged mizzenmast and both terms are present in Coflein. Even though SVM used the same training data that model was unable to predict it accurately. Precision, recall, and F1 scores for selected craft types are shown in Table 6, demonstrating that the model maintains strong performance beyond accuracy alone (average F1 = 0.88).

### Discussion

Our approach has enabled a rich dataset to be tagged by ship type, enhancing the information available to identify individual records. It is now possible to extract the entries for distinct types of ships much more easily. In the case of a shipwreck site—where the task is to identify known shipwreck events within a certain distance of the archaeological site to aid identification—this outcome reduces the amount of time required by a specialist to compile a list of relevant records. This streamlines the process of ascertaining an interpretation of a site and establish its significance. Even provision of a shortlist of possible related records would allow a greater degree of certainty when discussing archaeological sites in the marine zone. This could automate the manual process used for the identification of the shipwrecks like *Flower of Ugie* (Whitewright & Satchell, 2011) or *Ocean* (Pink & Whitewright, 2022). This in turn is an essential component to the management of heritage sites and the administration of marine developments.

One outcome that arose from conversations between the domain expert involved in the dataset and other maritime heritage specialists was that different heritage specialists apply some terms differently. It is therefore likely that the accuracy result for the model may always be lower than expected because domain experts see the tags differently. This means that human-in-the-loop is likely to remain an essential component of any Machine Learning application related to the archaeology of ships. Not only to ensure accuracy and use of correct terminology within the model but also to present the interpretation and reasoning behind different term choices to other domain specialists. In this regard the tools outlined here can be seen as a time-saving aid, and an interpretative assistant, not as a stand alone tool to interpret heritage data.

## Conclusion

The work presented here represents a contribution towards establishing a national collection. The datasets used, and the improvements made, will be the stepping off point for other National Heritage Records and other types of maritime heritage data. This paper has demonstrated that a text classification system can be used to enrich a heritage dataset and to extract key metadata terms for use in search. This is a significant development in the field of Maritime Archaeology, where no such system has yet been deployed on a dataset of this kind. This approach also offers a solid foundation for future studies on the application of limited labelled data.

The FSL approach demonstrated the model is a quick learner. As the FSL model performed with a decent level of accuracy, it can be concluded that it adapts to the domain with a small ‘training’ set. However, domain expertise is crucial for the model to provide reliable results, and human-in-the-loop remains an essential approach in this use case. Archaeology is a discipline that frequently deals with incomplete, messy datasets and domain expertise is essential to ensure that entities are surfaced correctly and the deployment of tools does not obscure other elements of the record. Utilising this approach our experiments within the domain of shipwreck archaeology show an accuracy of 83.85%. Essentially, this model is a time saving tool for a domain expert completing the heavy lifting of drawing out the type from a text-heavy description and streamlining the task of the archaeologist into one of correcting a tag rather than manually extracting each tag individually from widely varied text.

The results could improve further if labels were categorised into smaller subsets. Therefore reducing the number of labels the algorithm must go through in the first attempt. This could be an interesting route to explore with the FISH dataset. Any future development should target a larger dataset holding similar information. The record for England is the largest of the devolved heritage records in the U.K and has adopted the FISH Maritime Craft Thesaurus. It is also fully tagged with craft types. Deploying the FSL model on England’s Maritime Records represents an opportunity to take the findings from this study and develop the models further still. At its simplest, this will generate a much larger training dataset to explore the deployment of this tool.

These results demonstrate the potential of information extraction tools within archaeology. One of the biggest barriers to the use of these records is the time taken to extract meaningful information or to assemble shortlists of relevant entries for the study of a shipwreck site. The work done here has demonstrated the reduction in human-time required to extract the relevant information from a dense record. There is considerable potential for NLP tools to address other challenges in Maritime Data, a major area being how these datasets can be made more accessible and searchable. Current work using DNNs and NER provide a means to do this through ‘question-answering”. This has been used to deploy a model that can respond to direct queries with specific answers drawn from a corpus of data rather than providing a list of terms with a correct keyword or tagged with a certain field Suissa et al. (2022b). Drawing on the wider maritime heritage record to develop such a system would make a significant contribution to the utility of the UK’s Maritime Heritage Records. In this case, the tags the model extracted allow for enhanced search functionality, which can focus on specific components of the record, effectively refining search and not yet enabling a question answering method. That refined search functionality will assist prospection for sites and the interpretation of archaeological material. The other gain this process provides is the ability to quickly produce a high-level overview related to the numbers of different ship-types or show changes over time in the number and type of craft within Coflein. This was possible before, but required a significant investment of time to achieve. Such an overview can now be quickly extracted streamlining the process and opening routes to engage directly with the material the record points to.

## Data Availability

The data for this tool is available via Zenodo under the DOI 10.5281/zenodo.14888570 and accessible via the GitHub repository at: https://github.com/UnpathdWaters.

## Code availability

The complete codebase for this tool is available via the Unpath’d Waters GitHub repository at: https://github.com/UnpathdWaters
